# Occipital alpha-band brain waves when the eyes are closed are shaped by ongoing visual processes

**DOI:** 10.1038/s41598-022-05289-6

**Published:** 2022-01-24

**Authors:** Wiremu Hohaia, Blake W. Saurels, Alan Johnston, Kielan Yarrow, Derek H. Arnold

**Affiliations:** 1 School of Psychology, The University of Queensland, Brisbane, Australia; 2 School of Psychology, The University of Nottingham, Nottingham, UK; 3 School of Psychology, City University London, London, UK

**Keywords:** Neuroscience, Psychology

## Abstract

One of the seminal findings of cognitive neuroscience is that the power of occipital alpha-band (~ 10 Hz) brain waves is *increased* when peoples’ eyes are closed, rather than open. This has encouraged the view that alpha oscillations are a default dynamic, to which the visual brain returns in the absence of input. Accordingly, we might be unable to *increase* the power of alpha oscillations when the eyes are closed, above the level that would normally ensue when people close their eyes. Here we report counter evidence. We used electroencephalography (EEG) to record brain activity when people had their eyes open and closed, both before and after they had adapted to radial motion. The increase in alpha power when people closed their eyes was *increased* by prior adaptation to a broad range of radial motion speeds. This effect was greatest for 10 Hz motion, but robust for other frequencies (and especially 7.5 Hz). This discredits a persistent entrainment of activity at the adaptation frequency as an explanation for our findings. Our data show that the power of occipital alpha-band brain waves can be *increased* by motion sensitive visual processes that persist when the eyes are closed. Consequently, we suggest that the power of these brain waves is, at least in part, an index of the degree to which visual brain activity is being subjected to inhibition. This is increased when people close their eyes, but can be even further increased by pre-adaptation to radial motion.

## Introduction

One of the seminal findings of cognitive neuroscience has been that alpha-band activity in visual brain regions is *increased* when people close their eyes^1,2^. Long after this seminal observation, it was noted that neural spiking rates in cortex are *suppressed* when the power of alpha-band oscillatory activity is increased^3,4^. This has encouraged the idea that *decreases* in the power of alpha-band brain activity, when people open their eyes, are due to a *release from inhibition*^5^. This, in turn, prompted the suggestion that the level of alpha-band activity when people close their eyes is a *default* dynamic of the visual brain, to which it returns when there is no input^5^.

More recent investigations of alpha oscillations in the visual brain have focused on links between modulations of visual sensitivity, with the general finding that sensitivity tends to peak and trough in time with alpha oscillations in visual brain activity^6,7^. Also relevant are recent findings that visual sensitivity to inputs presented in one of two attended locations can also modulate in counter phase at an alpha frequency, peaking at one site while bottoming at the other—and vice versa^8,9^. These findings strengthen the view that alpha rhythms are a key dynamic of the human visual system, but they do not speak directly to the older foundational observation—that alpha-band oscillatory activity is enhanced when people close their’ eyes.

Rather than an idle-like state to which the visual system returns in the absence of input, the power of alpha-band oscillations when the eyes are closed might be a product of *ongoing* visual processes. This speculation is informed by the fact that human eye-lids are partially transparent. If you are in a lit environment you can demonstrate this to yourself. Close your eyes, and note the apparent brightness level. Then, additionally cover your eyes with your hand. When you do so, you should note that the apparent brightness level will *further decrease*. You can make your visual world darker still, even if you have closed your eyes—by additionally covering them with your hand. This not only demonstrates that your eyelids are transparent, it also demonstrates that visual processes are *ongoing* when your eyes are closed (otherwise you would not have noticed the further darkening of the visual world when you covered your closed eyes with your hand).

As visual processing is ongoing when you close your eyes, the power of alpha-band oscillations in occipital brain regions might be a product of visual processes that are ongoing, even when you close your eyes. We decided to see if these could be influenced by visual adaptation. Adaptation is a ubiquitous property of sensory coding, including visual processing^10^. Visual adaptation can be induced by prolonged exposure to a specific stimulus, or to a set of stimuli. It alters sensory activity for a protracted period, and changes perception^11^. Such changes are observed in motion^12^, orientation^13^, and shape perception^14^—to name just a few visual attributes. Visual adaptation is therefore a popular investigative tool to examine the computational processes underlying perception, leading to its colloquial title—the psychophysicist’s microelectrode^15^.

If the level of alpha power in occipital brain regions which prevails when people close their eyes is a default dynamic, to which the visual brain returns in the absence of input^16^, we might be unable to further *exaggerate* this dynamic via visual adaptation. If, however, the power of alpha-band oscillations is a product of ongoing visual processes, even when you close your eyes, we might be able to modulate these via motion adaptation.

To explore these possibilities, we used EEG to record brain activity from occipital sensors while people had their eyes open and closed, before and after they had adapted to radial motion. We had a small number of experienced observers adapt to a range of different speeds, across multiple testing sessions, so we could describe changes in eyes closed alpha power as a function of adapting speed. This suggested a range of effective frequencies. From these, we selected one (7.5 Hz) that did not coincide with the alpha rhythm of the visual brain (~ 10 Hz), and tested the robustness of our core finding with a larger group of volunteer participants. We found that the power of alpha-band oscillations, recorded by occipital sensors when the eyes are closed, can be *increased* by pre-adapting to radial motion.

## Results

### EEG data pre-processing and analyses

All analyses of data were conducted using custom MATLAB scripts using the FieldTrip toolbox^17^ and MATLAB’s in-built Fast-Fourier transform (FFT) command. All pre-processing was done offline. Data were high-pass (2 Hz), low-pass (60 Hz), and band-stopped filtered (49–51 Hz) using a 6th order Butterworth filter with a twopass direction. Blink artefacts were removed via an independent-components analysis (ICA). Data were re-referenced to the volume average voltage (from across all 64 channels), and then epoched into six-second segments, separately for eyes open and closed trials, and for the baseline and adaptation trials.

For each participant, spectra were calculated on data from all 64 channels. For our first data sample (N = 6), individual peak-frequency was estimated as the frequency (within the range 3–17 Hz) at which baseline eyes-closed power was greatest (M =  ~ 10 Hz) on eyes closed trials. Because alpha oscillations are stronger when the eyes are closed, we reasoned that individual peak-alpha frequency (IPAF) estimates would be more accurate if taken from these data.

**Figure 1:**
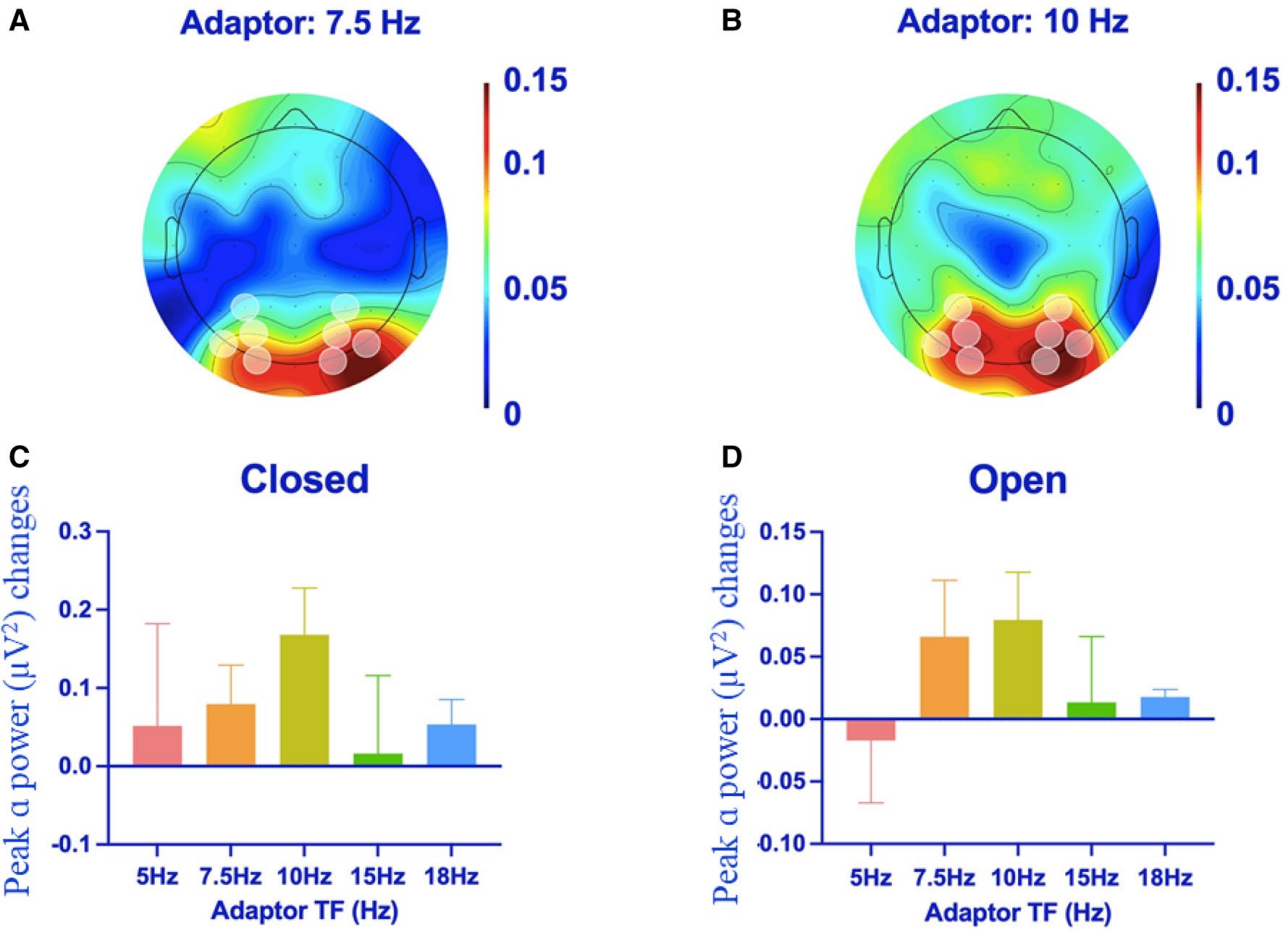
Results for experienced participants. Positive values indicate an *increase* in IPAF power (~ 10 Hz) on adaptation trials, relative to baseline estimates. (**A**) Topographical distribution of peak alpha changes when the eyes were closed, after adapting to 7.5 Hz rotations. Left and right hemisphere occipital sensors are marked. (**B**) As for (**A**), but for the 10 Hz adaptor. (**C**) Peak-alpha changes (adapted–baseline) when the eyes were closed as a function of adapting temporal frequency. Error bars denote ± 1SEM. (**D**) As for (**C**), but for eyes-open data. Note, data in (**C**,**D**) are plotted on different scales. Lastly, *a.u* denotes arbitrary units.

Differences between adaptation and baseline conditions were calculated for each individual, separately for eyes open and closed trials. To illustrate the spatial distribution of adaptation-driven changes, we have plotted changes in IPAF power at all electrodes, averaged across all experienced participants. These illustrations relate to changes after adapting to 7.5 Hz (see Fig. 1A) and to 10 Hz (Fig. 1B) radial motions. Note that post-adaptation, increases in alpha power seem to cluster in recordings from occipital sensors.

For experienced participants, adaptation-induced changes in alpha power recorded by occipital sensors (O1, O2, PO3, PO4, PO7, PO8, P3, P4; see Fig. 1A,B) are illustrated in Fig. 1C (for recordings taken when the eyes were closed) and 1D (for recordings taken while the eyes were open). These data suggest a broadly tuned function, maximal for ~ 10 Hz adaptation, but also seemingly robust for 7.5 Hz adaptation. Of these frequencies, we chose 7.5 Hz as the adaptation frequency for volunteer student participants, as this frequency does not coincide with average peak-alpha frequency in our sample (~ 10 Hz). So using this adaptation frequency avoids a modulated frequency tag, at the adaptation frequency, from providing a credible explanation for changes in peak-alpha power.

**Figure 2:**
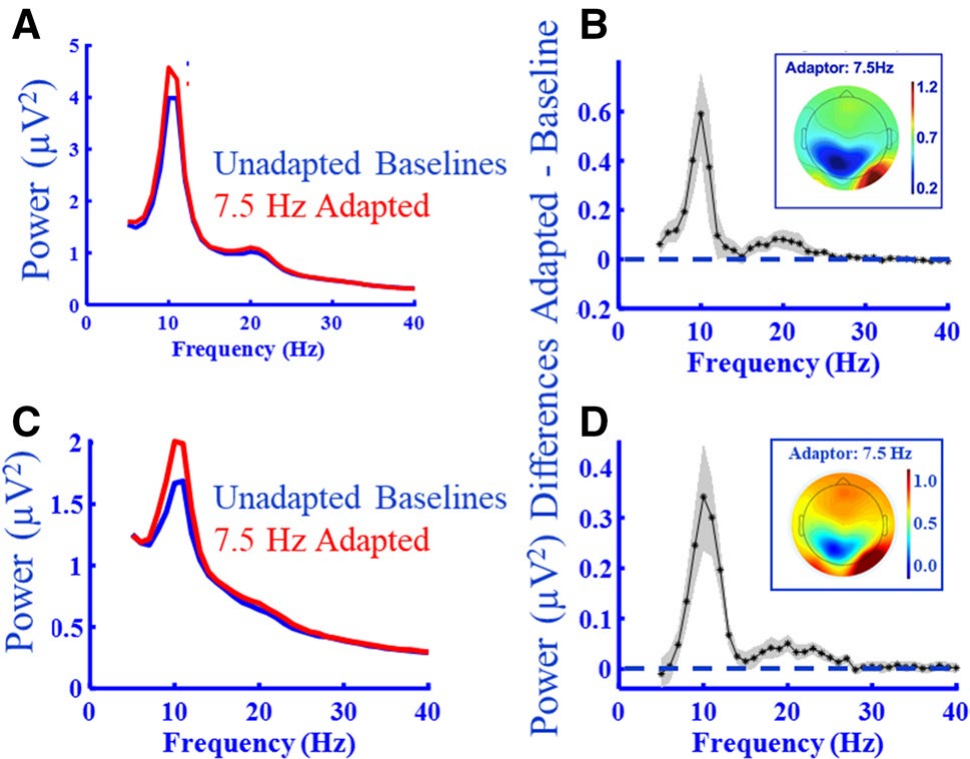
Experiment 2 results. (**A**) Grand-averaged power, prior to adaptation (blue data) and after adaptation to 7.5 Hz motion, as a function of oscillatory frequency. Both datasets were recorded while the eyes were *closed*, from occipital sensors: O1, O2, PO3, PO4, PO7, PO8, P3, P4. (**B**) Grand-average power differences (adapted–unadapted baseline) as a function of oscillatory frequency. The shaded region depicts ± 1SEM. Positive values signify greater power post adaptation. (**B**-inset) Group-level topographical map of adaptation driven IPAF power changes (~ 10 Hz, see Fig. 5) recorded during eyes closed periods. (**C**,**D**) As for (**A**,**B**), but for data recorded while the eyes were *open*.

The following analysis details apply to our larger sample of volunteer student participants (N = 25), who provided the basis for formal testing of our principal finding—that motion adaptation enhances eyes-closed alpha power. Data for these analyses were taken from occipital sensors (as marked in Fig. 1A,B). For these participants, absolute power differences, between baseline and 7.5 Hz adapted eyes-closed data, are plotted in Fig. 2 as a function of oscillatory frequency. These data suggest that 7.5 Hz motion adaptation had a maximal impact on intrinsic power at ~ 10 Hz—justifying the selection of this adaptation frequency. The distribution of these changes was clustered at occipital sensors (see Fig. 2 inserts).

**Figure 3:**
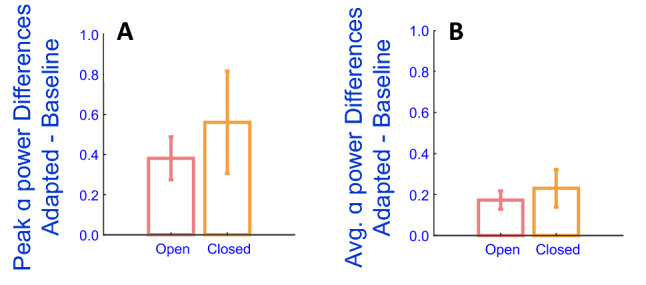
Experiment 2 results. (**A**) Average IPAF power changes by condition. These data were first averaged across occipital sensors for each individual, then across individual participants. Error bars depict ± 1SEM. (**B**) Adaptation-induced power changes, averaged across alpha-band frequencies by condition. Error bars depict ± 1SEM.

For the following analyses IPAFs were estimated separately for Eyes Closed and Eyes Open datasets, from estimates calculated from data recorded by occipital sensors, which were then separately averaged for both baseline and adapted trials—to protect against the possibility that any differences might be due to changes in peak alpha frequencies across experimental/adaptation conditions^18–20^. These data were subjected to a 2 (Adapted/Unadapted Baseline) × 2 (Eyes Open/Closed) repeated measures ANOVA on conditional estimates of occipital IPAF power. We observed a main effect of Adaptation, wherein IPAF power was greater post 7.5 Hz radial motion adaptation relative to unadapted baseline estimates (F_1,24_ = 70.81, *p* < 0.001, η^2^ = 0.747; see Fig. 3A). Similarly, there was a main effect of Eyes Open / Closed, such that IPAF power was greater when the eyes were closed (F_1,24_ = 11.85, *p* = 0.002, η^2^ = 0.331; see Fig. 3A). There was no interaction between these main effects (F_1,24_ = 0.47, *p* = 0.500).

The pattern of results depicted in Fig. 3A did not rely on selecting IPAFs for analyses, as this pattern persisted for a further analysis wherein we averaged conditional data across *all* alpha band frequencies (~ 7–14 Hz; see Fig. 3B) prior to conducting an ANOVA (as opposed to selecting IPAFs). For these analyses, oscillatory power estimates were individually averaged across occipital electrodes, and then across all alpha frequencies—separately for the eyes-closed and eyes-open conditions. Data were then subjected to a repeated measures ANOVA, as above. Again, we found that alpha power was greater post 7.5 Hz radial motion adaptation (F_1,24_ = 64.05, *p* < 0.001, η^2^ = 0.727; see Fig. 3B) and when the eyes were closed (F_1,24_ = 10.11, *p* = 0.004, η^2^ = 0.296; see Fig. 3B), and there was no interaction between these effects (F_1,24_ = 0.49, *p* = 0.489).

It is also important to note that delays between being cued to close your eyes, and participants actually closing their eyes, were equivalent across baseline (average 0.99 s, S.D. 0.13) and adaptation (average 0.96 s, S.D. 0.14; paired t_24_ = 0.84, p = 0.389) trials—as determined by visual inspection of raw (un-preprocessed) EEG recordings from frontal sensors.

To avoid any possibility that adaptation might impact baseline estimates of alpha power, adaptation trials always followed a preliminary block of baseline trials. This, however, raises the question of whether time elapsed in the experiment, fatigue, or modulations in arousal, might explain our results^21^. To address this possibility, we calculated differences in occipital IPAF power (as per the analysis sequence described for data in Fig. 3A) as a function of time elapsed during each 6-s testing period. For this analysis, estimates of occipital IPAF power were calculated from the first second of each 6-s baseline testing period, and these were subtracted from estimates calculated for the matching period of adapted trials, and so on for each second of our 6-s testing periods. If increases in alpha power were dirven by adaptation, we would expect these to be greatest at the start of each testing period (at times nearest adaptation) and then to decline. If, however, increased alpha power were due to accumulating fatigue, we would either expect these effects to increase as trials progressed (due to more time having elapsed), or to show no variance (as time elapsed on individual trials might be inconsequential relative to the overall amount of time elapsed during an experimental session).

**Figure 4:**
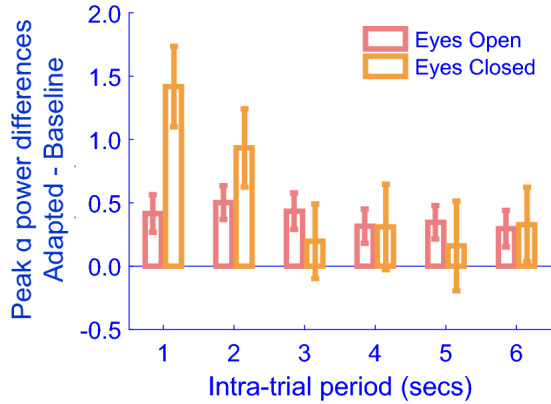
Average occipital IPAF power differences (y-axis) as a function of time during testing periods (x-axis). Data are difference scores, with positive values denoting greater occipital alpha power post-adaptation. Error bars depict ± 1SEM. These data show that Eyes-Closed adaptation effects were declining within each 6-s testing period.

We conducted a 2 (Eyes: Open, Closed) × 6 (Time—in seconds) repeated measures analysis of variance (ANOVA) on intra-trial changes in occipital IPAF power differences (Adapted–Baseline). There was a significant main effect of Time (F_5,120_ = 5.16, *p* < 0.001, η^2^ = 0.177; see Fig. 4). Moreover, there was a significant interaction between Eyes Open/Closed and Time of testing (F_5,120_ = 3.7, *p* = 0.004, η^2^ = 0.134; see Fig. 4), with Eyes-Closed occipital IPAF power being relatively greatest for Eyes Closed trials at the begining of testing periods (at a time nearest adaptation), but this effect declined until power estimates were reasonably well-matched across conditions by the end of testing periods. This characterisation of these data are consistent with significant linear (F_1,24_ = 4.26, *p* = 0.05, η^2^ = 0.151) and quadratic (F_1,24_ = 7.45, *p* = 0.012, η^2^ = 0.237) interactions between the main effects of Eyes (Open or Closed) and Time. No other order of interaction was significant (minimum *p* = 0.134).

Intra-trial reductions in Eyes-closed IPAF power differences suggest that these are likely driven by adaptation, as opposed to a pure consequence of fatigue (which should have tended to increase rather than decline as a function of time). Occipital IPAF power differences did not, however, dissapear entirely by the end of our 6-s testing periods. This leaves open the question of whether residual differences were due to a persistent adaptation effect, or to accumulating fatigue. To assess which of these explanations was more plausible, we examined how our putuaive adaptation effects varied across experimental sessions. Specifically, we calculated separate difference scores between occipital IPAF power estimates taken from baseline trials and from pairs of adapted trials, conducted either Early (the first two adapted trials for each experimental condition) Late (the last two adapted trials for each condition) or near the Mid-point (middle two adapted trials for each condition) of experimental sessions. If IPAF power differences were related to accumulating fatigue, we would expect these to increase as a function of time during experimental sessions. If, however, differences were related to adaptation, we would expect these to be apparent from the earliest adaptation trials, which followed immediately after baseline trials, and to be relatively constant thereafter.

**Figure 5:**
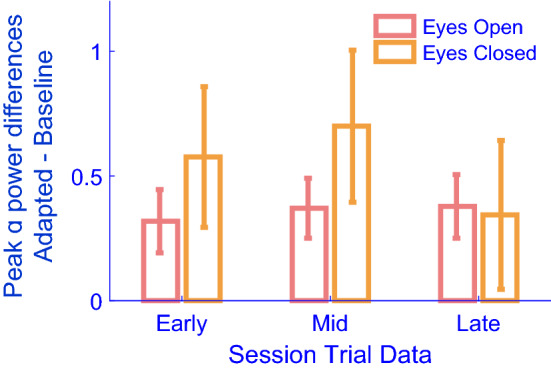
Average individual peak-alpha frequency power differences (y-axis) as a function of time during the experimental session (x-axis). Differences are calculated between baseline data and the first 2 adapted trials (early), two adapted trials from the mid-point of experimental sessions (mid) and the final two adapted trials (late). Error bars depict ± 1SEM. These data show that the effects of adaptation were evident from the first adapted trials, and were relatively constant across each experimental session.

We conducted a 2 (Eyes: Open/Closed) × 3 (Time in experimental session, Early, Mid or Late) repeated measures analysis of variance (ANOVA), examining changes (Adapted–Baseline) in occipital IPAF power. This did not reveal any significant main effects or an interaction. Moreover, we conducted a paired t-test examining occipital IPAF power differences (averaged across conditions) calculated from Early adaptation and from baseline trials. This revealed a robust difference for the first two adaptation trials completed for each experimental condition (t_24_ = 2.99, p = 0.006, 95% CIs 0.14–0.76; see Fig. 5). As these trials followed immediately after baseline trials, this analysis provides strong evidence that differences were due to adaptation, rather than accumulating fatigue.

**Figure 6:**
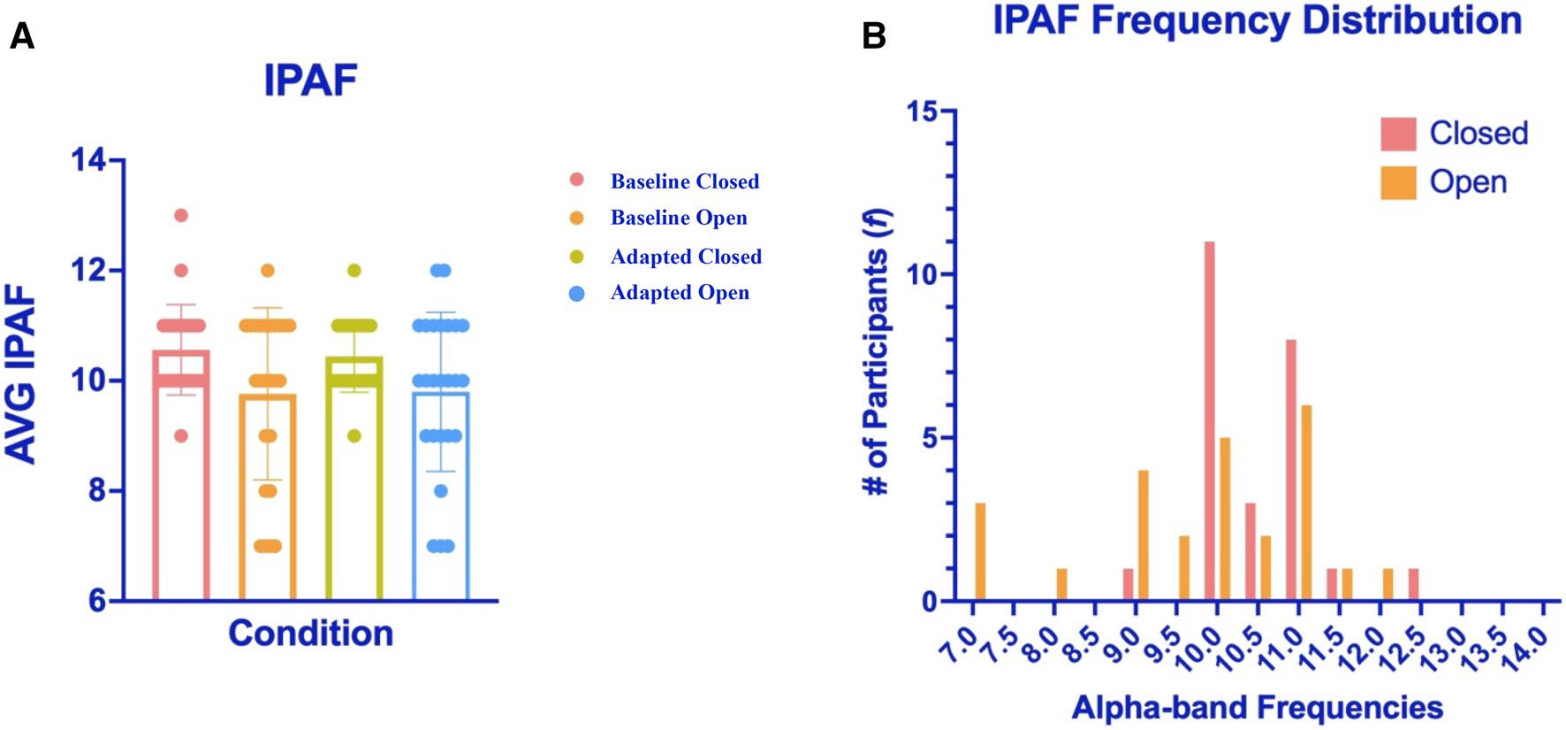
Results of a repeated measures ANOVA of IPAF by condition. (**A**) Whole-group averaged IPAF for baseline and adaptation blocks, separated by eyes-open and closed conditions. Error bars denote ± 1SD. (**B**) Frequency histogram of IPAF for eyes-closed (pink) and eyes-open (orange) data. Baseline and adaptation data are combined, the max power was obtained for each individual, for both eyes-open and eyes-closed conditions separately.

As we have mentioned above, IPAFs can differ when the eyes are open rather than closed^21^. To see if this has impacted our data, we estimated IPAFs for each participant, separately for data recorded when the eyes were open and closed—at baseline and after adaptation to 7.5 Hz radial motion (see Fig. 6A,B). These data were then subjected to a repeated measures ANOVA. While this analysis revealed a significant main effect of testing condition (Eyes Open / Closed; F_1,24_ = 7.87, *p* = 0.010, η^2^ = 0.25), with higher IPAFs when the eyes were closed (M = 10.50) rather than open (M = 9.80), there was no main effect of testing period (Baseline vs Adaptation; F_1,24_ = 0.13, *p* = 0.723, η^2^ = 0.005), or an interaction between testing condition and period (F_1,24_ = 0.41, *p* = 0.527, η^2^ = 0.017; see Fig. 6A). Figure 6B displays a histogram of IPAFs for eyes-closed and eyes-open data separately, collapsed across baseline and adaptation trials. Overall, these data rule against the possibility that our adaptation effects (i.e. enhanced alpha power) can be attributed to having contrasted data characterised by different IPAFs—as there is no evidence that IPAFs were changed by adaptation. Also, bear in mind that our effects were robust even when we averaged data across all alpha frequencies (see Fig. 3B), and when we used conditional specific IPAF estimates for analyses (see Fig. 3A).

## Discussion

A seminal finding of cognitive neuroscience is that alpha-band activity in visual brain regions is *increased* when people close their eyes^1^. We have shown that this effect can be further *increased* by pre-adapting to radial motion. This effect appears to be broadly tuned for temporal frequencies of about 10 Hz, but is robust after adaptation to 7.5 Hz radial motion (see Figs. 1 and 2). We note that the power of peak alpha-band activity was increased by pre-adaptation to radial motion, regardless of whether the eyes were open or closed during testing. For most of our analyses, there was no interaction between adaptive state and whether the eyes were open or closed—the only exception being our analyses of power changes during trials (see Fig. 4, and related analyses). The most appropriate interpretation of our data, therefore, is that radial motion adaptation serves to enhance the power of alpha-band oscillatory activity, both when the eyes are open and closed.

To avoid adaptation-related contamination of baseline data, radial motion adaptation *always* followed baseline measures. This precaution, however, raises the possibility that our adaptation effects might have been due to differences in arousal^21^, rather than to adaptation. A number of observations speak against this possibility. First, our data suggest some degree of specificity, with maximal adaptation effects after ~ 10 Hz adaptation. We would not expect arousal to be selective for adaptation frequency. Second, on adaptation trials increases in occipital eyes-closed IPAF power were greatest during the first second of each 6-s testing period, and then declined (see Fig. 4). This is consistent with an adaptation effect that is greatest nearest in time to adaptation, which then reduces in magnitude. We would expect an opposite trend if increased alpha power were instead due to accumulating fatigue. Third, we found no evidence that adaptation effects were subject to a substantive change as a function of time elapsed within an experimental session (see Fig. 5). Moreover, adaptation effects were apparent for the first 2 adapted trials for each experimental condition, which were conducted immediately following baseline trials (see Fig. 5). Finally, we note that the experimental task was not demanding (passive viewing of a screen), and testing sessions were brief (approximately 30 min), so we would not expect participants to be much fatigued by our experiment.

**Figure 7:**
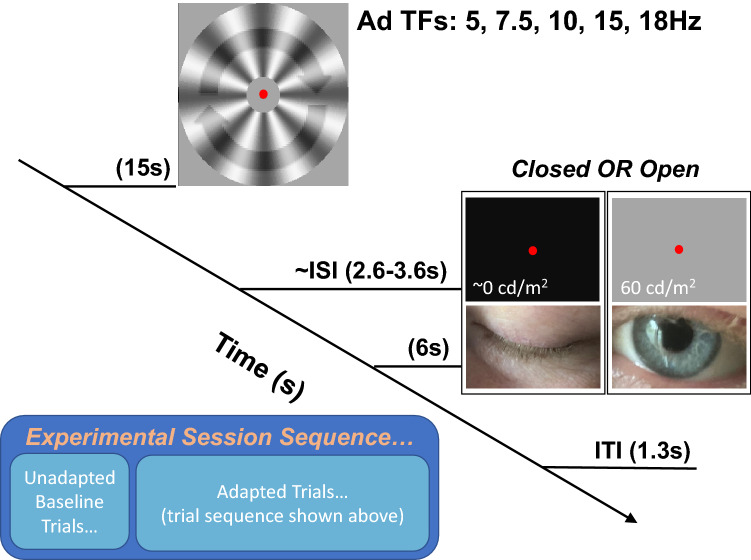
Graphic depicting the experimental protocol. On each adapted trial, participants were exposed to radial motion for 15 (experienced participants) or for 20 (volunteer student participants) seconds, animated at one of five adapting frequencies (5, 7.5, 10, 15, or 18 Hz). After adaptation participants could be prompted to close their eyes (by a low-pitched tone) or simply had to keep them open while fixating centrally. A variable delay (of 2.6–3.6 s) followed. Brain activity was then recorded for 6 s, before the participant was prompted to re-open their eyes (if they had been closed) by a high-pitched tone. There was then a fixed inter-trial-interval (ITI) before the adaptor was re-presented, during which people finished re-opening their eyes if this was necesary. The procedure for baseline trials was similar, but excluded adaptation periods. Insert: all unadapted baseline trials *had* to precede adaptation periods (otherwise, they would not be unadapted), so these were conducted at the beginning of an experimental session, before any adaptation.

Our data suggest that the effects of motion adaptation were long-lasting—still evident in the final second of our 6 s test periods across both experimental conditions (see Fig. 4). This was not unexpected. We used relatively protracted adaptation periods (20 s for volunteer student participants, see Fig. 7) on each and every adaptation trial, and the consequences of adaptation are known to be protracted when people close their eyes^22^. Consequently, we took the precaution of conducting baseline analyses on data recorded *prior* to any adaptation, rather than from periods immediately preceding adaptation on each trial. We had assumed data recorded during periods in-between adaptation trials would be at least partially adapted, and our data seem to validate this precaution (see Fig. 4).

One potential criticism of our core finding is that the enhanced occipital alpha power could be explicable in terms of a post-adaptation refractory period^23,24^. The proposal would be that neurons that had been responsive to the adaptor would hyperpolarise for a period after the offset of the adaptor, allowing for a greater power of oscillatory occipital alpha activity while spontaneous firing is suppressed. This suggestion would seem to uphold a definition of the ‘refractory’ period that is specific to EEG studies^23^. In electrophysiological studies the refractory period refers to a time following an action potential during which it is either impossible to generate more action potentials (the absolute refractory period), or during which action potentials are greatly suppressed (the relative refractory period^24^). However, these periods are measured in the order of milliseconds—far too short to account for the persistent enhancement of oscillatory occipital alpha power we have observed (we only began to analyse data ~ 3.1 s post adaptation, see Fig. 7).

It is possible that the suggestion that our data might be accounted for via a post-adaptation refractory period reflects the use of different terminology to describe a common process in different fields of research. What some EEG researchers describe as a refractory period might be described as a persistent impact of sensory adaptation by fMRI researchers, electrophysiologists and psychophysicists^24^. Indeed, there is an extensive literature examining the rates at which different visual mechanisms adapt, and then recover (for a review, see^11^). For instance, the reasonably protracted adaptation periods we have used (20 s on each trial for volunteer student participants) are known to simultaneously induce adaptation effects across multiple visual mechanisms, which both develop and then recover to unadapted states across different characteristic timecourses^11^. So, visual adaptation should be understood as having triggered processes that are ongoing until the consequences of adaptation have extinguished.

Another limitation of our data is that we were unable to attempt to estimate the source of alpha oscilations, as we did not conduct structural fMRI scans for our participants, and alpha power recorded by occipital sensors can arise from multiple sources^18,19,25^. This is an obvious limitation of our study that should be addressed in future investigations.

Long after the eyes closed effect^1,2^ was first reported, it was shown that during periods of increased alpha-band synchronisation neural spiking in cortex is suppressed^3,4^. This encouraged the idea that decreases in occipital alpha power when people open their eyes is due to a release from inhibition^5^. This, in turn, prompted the suggestion that the level of alpha-band activity when people close their eyes represents a *default* dynamic of the human visual brain, which is disrupted when people open their eyes^5^. Our data question this view.

Rather than an idle-like state to which the visual system returns in the absence of input, our data suggest that eyes-closed alpha oscillations are, at least in part, a product of ongoing visual processes. Otherwise they should not have been impacted by visual adaptation, which modulates visual processing^11^. We feel the classic account of the eyes-closed alpha effect misconstrues eyes-closed conditions as periods when all visual input has been eliminated and visual processes have ceased. Closing your eyes does not instigate a cessation of visual processing, as should be apparent if you try the simple demonstration outlined in our introduction.

One important facet of our data is that we were able to use adaptation to 7.5 Hz radial motion to increase alpha-band (~ 10 Hz) oscillatory power in visual brain regions. This is interesting, both because it precludes an ongoing stimulus-driven frequency tag from providing a viable interpretation of our data^24^, and because it might provide some insight into the tuning characteristics of the mechanisms that generate the alpha rhythm. While humans can perceive a broad range of different speeds, evidence suggests this capacity relies on systems-level mechanisms that are broadly tuned to a small number of temporal frequencies. These have been estimated as being 0 Hz (static), ~ 10 Hz, and ~ 18 Hz^26^. It is intriguing that one of these broadly-tuned mechanisms (10 Hz) would be responsive to our chosen adaptor (7.5 Hz). Indeed, the broadly tuned profile suggested by our data (Experiment 1) would be consistent with this temporal-frequency tuned channel mediating the effects we have discovered. Another potentially relevant observation is that adult human contrast sensitivity tends to be maximal for stimuli modulated at ~ 10 Hz, as opposed to near newborns, who are maximally sensitive to static inputs^27^. There are clearly large numbers of neurons in adult human visual cortex that are (maximally) responsive to inputs animated at ~ 10 Hz, but which are also responsive to other dynamics (including 7.5 Hz). Our data suggest that adapting these can transiently increase the power of oscillatory occipital brain activity—including when you subsequently close your eyes.

While our investigation has targeted the seminal finding—that occipital alpha power is enhanced when the eyes are closed, our data may have relevance to other contemporary investigations. For instance, visual sensitivity has been shown to fluctuate at an alpha frequency in tandem with visual brain activity^6,7^. Our data encourage an investigation to see if this tendency can be enhanced by adaptation. Similarly, sensitivity to inputs in one of two spatially distributed positions can vary in counter phase at an alpha frequency^8,9^. Could this tendency be exaggerated by adaptation?

It has been assumed that alpha is a default dynamic of the human visual system, to which it returns when visual input is removed. Our data caution against this interpretation. They establish that the power of eyes closed alpha-band activity can be further *increased* by pre-adapting to radial motion. This shows that eyes closed alpha-band oscillations are, at least in part, a product of activity that interacts with processes that can be changed by adaptation to radial motion. Moreover, this influence can be seen to gradually reduce—even when the eyes are closed. What could explain these observations?

One possibility is that inhibition in visual brain regions *increases* relative to baseline levels when the eyes are closed, but this effect can be further increased by pre-adapting to visual motion. Experiments involving single cell recordings and spatial attention have shown that spatially targeted inhibition of visual signalling is associated with an increase in alpha power^28–33^. The power of alpha-band oscillations in visual brain regions is also enhanced when attention is directed to audio inputs^31^—consistent with a general suppression of visual processing when visual input is less attended (for a review, see^33^). The increase in alpha power across visual brain regions when people close their eyes might be similar—attention might be directed to other sensory inputs, including audition, and to memories^33,34^, resulting in visual processing being generally suppressed via inhibition. Our data suggest that visual adaptation can increase this effect, perhaps by reducing the spontaneous firing rates of large numbers of visual neurons that are responsive to dynamic inputs^26,27^. This could make visual brain regions more susceptible to inhibition. Obviously, these proposals are speculative, and will require further investigation.

The firm conclusion that can be drawn from our data is that the power of alpha-band oscillations, in occipital brain regions when people close their eyes, is at least in part a product of visual states/processes that can be modified by motion adaptation. This encourages an alternate interpretation relative to the classic explanation of the eyes closed occipital alpha effect^1,2^. We suggest that alpha-band oscillations in visual brain regions scale with a process that is ongoing when people close their eyes. Specifically, rather a default dynaic of the visual brain, our data are consistent with the view that occipital alpha-band oscillations index the degree to which visual brain activity is being subjected to inhibition^33,34,36^, which tends to be increased when people close their eyes, but which can be increased futher still by having people pre-adapt to radial motion.

## Methods

### Ethics

Ethical approval for our experiments was obtained from the University of Queensland’s (UQ) Ethics Committee, and all experimental tasks were performed in accordance with the UQ guidelines and regulations for research involving human participants. Each participant provided informed written consent to participate in the study and were made aware that they could withdraw at any moment from the study without prejudice or penalty.

### Participants

Six lab members (5 male, M_age_ = 27.5) volunteered to participate as *experienced* observers who could be tested repeatedly. Three of these were authors (WH, BWS, DHA). To assess the robustness of findings suggested by data from these participants, we recruited a further 25 students from a research participation pool in the School of Psychology at The University of Queensland (15 male, M_age_ = 19). All of these student participants were naïve as to the purpose of the experiment, supplied informed consent to participate, and received course-credit in exchange for participation. All participants reported having normal, or corrected-to-normal visual acuity (we asked them to wear glasses/contact lenses if they would normally do so while reading).

### Design

A 2 (Eyes: Open, Closed) × 5 (Adaptor temporal frequency: 5, 7.5, 10, 15, 18 Hz) repeated measures design was used in Experiment 1. Preliminary baseline recordings were taken prior to adaptation blocks. This was essential so as to avoid any possibility of adaptation impacting baseline estimates. In Experiment 2, the design was identical, except one adaptor temporal frequency was administered.

### Stimuli and apparatus

Testing took place in a darkened room. A chinrest ensured a constant viewing distance (57 cm). During all eyes-open periods (including adaptation), participants fixated a central red dot, with a diameter subtending 0.4 degrees of visual angle (dva) at the retina. Stimuli were presented on an ASUS VG248QE 3D Monitor (1920 × 1080 pixels, refresh rate: 60 Hz), driven by a Cambridge Research Systems ViSaGe stimulus generator and custom MATLAB R2015b^17^ software. A Tucker-Davis Technologies (TDT) Audio Workstation was used to produce audio cues, emitted diotically at supra-threshold level by speakers on either side of the testing display. The adaptor consisted of a sine-wave luminance-modulated radial grating (8 cycles; radial frequency: 4/πc/rad; contrast: 50%), presented within an annulus with an outer diameter subtending 29 dva and an inner annulus subtending 2 dva. EEG data were recorded using the Biosemi International ActiveTwo system. Electrodes (64 Ag/AgCl) were placed according to the extended international 10–20 system and digitised at a 1024 Hz sample rate with 24-bit analog–digital conversion. The standard BioSemi reference and ground electrodes were used during recording.

Adaptor rotation direction was unidirectional, and counterbalanced across both experienced and student participants. For experienced participants, adapting speeds (5, 7.5, 10, 15, and 18 Hz) were adjusted for different blocks of trials. For these participants, blocks of trials for the different adaptors were conducted on different days, and in a pseudo random order. Baseline recordings were administered each day of testing. Student participants completed a single experimental session, adapting to 7.5 Hz rotation.

### Procedure

Each experimental session began with preliminary baseline recordings, 48 trials for experienced participants, and 32 trials for volunteer participants. In each case, half of these involved testing periods with the eyes open, and half with the eyes closed. All trials involved 6 s testing periods, during which we recorded brain activity. Baseline trials were conducted first to prevent any possibility of baseline data being impacted by adaptation, but this confounds adaptive state with time elapsed in the experimental session. This confound was, however, constant across all adaptation frequencies, so it cannot explain any differential impact of adaptation. Additional control analyses were conducted to address this issue.

On baseline trials, participants were cued to close and open their eyes, by low- and high-pitched audio tones. Subjects were given ample time (~ 2.6 to 3.6 s) to close their eyes, or to remain fixated. This was necessary both because it took participants some time to close their eyes on relevant trials, and because we wanted to have a sufficient delay in-between people closing their eyes and the onset of brain activity recordings in order to avoid recordings being contaminated by transients associated with the sudden darkening of the visual field when people closed their eyes. During eyes-closed periods a uniform dark display was presented in front of the participant. During eyes-open periods participants fixated the red dot in the middle of an otherwise grey display. In each case recordings of brain activity were then taken for 6 s, after which time the participant was cued to re-open their eyes (if they had been closed). There was then a fixed ITI of 1.3 s (allowing sufficient time to re-open the eyes when this was necessary) before the next procedure began.

Experimental sessions also included 48 (experienced participants) or 32 (volunteer student participants) adaptation trials. These were alike baseline trials, except that each began with the presentation of an adapting stimulus, for either 15 s (experienced participants) or 20 s (for our more numerous student volunteer participants). The adapting stimulus then disappeared, at which point participants were either cued to close their eyes, or there was no cue (so participants’ eyes remained open, see Fig. 7 for a graphic depicting the experimental protocol). There was then a variable delay (of 2.6 to 3.6 s) to allow participants sufficient time to close their eyes when this was necessary. A full experimental session therefore included either 96 (experienced participants) or 64 (volunteer student participants) individual trials.

### Informed consent

All images and figures in the manuscript belong to the authors who consent to these being published.

## Data Availability

All EEG data and analysis scripts for this project will be made available via UQeSpace https://espace.library.uq.edu.au/.
